# Encoding laboratory testing data: case studies of the national implementation of HHS requirements and related standards in five laboratories

**DOI:** 10.1093/jamia/ocac072

**Published:** 2022-05-25

**Authors:** Raja A Cholan, Gregory Pappas, Greg Rehwoldt, Andrew K Sills, Elizabeth D Korte, I Khalil Appleton, Natalie M Scott, Wendy S Rubinstein, Sara A Brenner, Riki Merrick, Wilbur C Hadden, Keith E Campbell, Michael S Waters

**Affiliations:** Deloitte Consulting LLP, Washington, District of Columbia, USA; Office of the National Coordinator for Health Information Technology, Washington, District of Columbia, USA; U.S. Food and Drug Administration, Silver Spring, Maryland, USA; Deloitte Consulting LLP, Washington, District of Columbia, USA; Deloitte Consulting LLP, Washington, District of Columbia, USA; Deloitte Consulting LLP, Washington, District of Columbia, USA; Deloitte Consulting LLP, Washington, District of Columbia, USA; Deloitte Consulting LLP, Washington, District of Columbia, USA; U.S. Food and Drug Administration, Silver Spring, Maryland, USA; U.S. Food and Drug Administration, Silver Spring, Maryland, USA; U.S. Department of Health and Human Services, Silver Spring, Maryland, USA; Association for Public Health Laboratories, Silver Spring, Maryland, USA; University of Maryland, College Park, Maryland, USA; U.S. Food and Drug Administration, Silver Spring, Maryland, USA; U.S. Department of Veterans Affairs, Bend, Oregon, USA; U.S. Food and Drug Administration, Silver Spring, Maryland, USA

**Keywords:** health information interoperability, clinical laboratory information systems, public reporting of healthcare data, Logical Observation Identifiers Names and Codes

## Abstract

**Objective:**

Assess the effectiveness of providing Logical Observation Identifiers Names and Codes (LOINC®)-to-In Vitro Diagnostic (LIVD) coding specification, required by the United States Department of Health and Human Services for SARS-CoV-2 reporting, in medical center laboratories and utilize findings to inform future United States Food and Drug Administration policy on the use of real-world evidence in regulatory decisions.

**Materials and Methods:**

We compared gaps and similarities between diagnostic test manufacturers’ recommended LOINC® codes and the LOINC® codes used in medical center laboratories for the same tests.

**Results:**

Five medical centers and three test manufacturers extracted data from laboratory information systems (LIS) for prioritized tests of interest. The data submission ranged from 74 to 532 LOINC® codes per site. Three test manufacturers submitted 15 LIVD catalogs representing 26 distinct devices, 6956 tests, and 686 LOINC® codes. We identified mismatches in how medical centers use LOINC® to encode laboratory tests compared to how test manufacturers encode the same laboratory tests. Of 331 tests available in the LIVD files, 136 (41%) were represented by a mismatched LOINC® code by the medical centers (chi-square 45.0, 4 df, *P* < .0001).

**Discussion:**

The five medical centers and three test manufacturers vary in how they organize, categorize, and store LIS catalog information. This variation impacts data quality and interoperability.

**Conclusion:**

The results of the study indicate that providing the LIVD mappings was not sufficient to support laboratory data interoperability. National implementation of LIVD and further efforts to promote laboratory interoperability will require a more comprehensive effort and continuing evaluation and quality control.

## BACKGROUND AND SIGNIFICANCE

### Motivation

Healthcare laboratory data exchange requires that laboratories reproducibly encode their test data using industry coding standards. For laboratory data, appropriate use of Logical Observation Identifiers Names and Codes (LOINC®)^1^ and Systematized Nomenclature of Medicine Clinical Terms (SNOMED CT®),^2^ is essential to ensure tests and results are accurately and reliably described within electronic health records (EHR), laboratory information systems (LIS), and public health reports.

The absence of laboratory semantic interoperability for *in vitro* diagnostic (IVD) data has been cited as a significant impediment to overall public healthcare.^3^^,^^4^ Concerns regarding the interoperability and reliability of LOINC® between organizations have inspired several scholarly and practical efforts to champion and facilitate laboratory data exchange.^5–16^ The erosion of accuracy for IVD test data due to interoperability failures can have patient safety consequences and impede timely access to and analysis of lab data on a nationwide scale.^17^ The problems with interoperability of laboratory data were brought to national attention by the COVID-19 pandemic, which highlighted an inability to estimate disease incidence and difficulties associated with monitoring testing.^18^ Section 18115 of the Coronavirus Aid, Relief, and Economic Security (CARES) Act (P.L. 116-136) requires laboratories to report COVID-19 test results to the United States (U.S.) Health and Human Services (HHS) Secretary.^19^

In order to address laboratory data interoperability challenges, the U.S. Food and Drug Administration (FDA), IVD Industry Connectivity Consortium (IICC), and Medical Device Innovation Consortium (MDIC) sponsored SHIELD (Systemic Harmonization and Interoperability Enhancement for Laboratory Data), a public–private partnership focused on improving the quality, interoperability, and portability of IVD data within and between institutions. The SHIELD collaborative is a multi-agency/stakeholder network consisting of FDA, U.S. Centers for Disease Control and Prevention (CDC), U.S. National Institutes of Health (NIH), the Office of the National Coordinator for Health Information Technology (ONC), U.S. Centers for Medicare and Medicaid Services (CMS), U.S. Department of Veterans Affairs (VA), IVD manufacturers, EHR vendors, laboratories, College of American Pathologists, standards developers, Pew Charitable Trusts, National Evaluation System for health Technology, and academia.^4^

SHIELD developed the LIVD (LOINC® to IVD) mapping specification for IVDs with Emergency Use Authorization (EUA) by FDA for SARS-CoV-2 diagnostic use, which harmonizes how IVD test information is represented using laboratory data standards.^20^ LIVD focuses on describing the same laboratory test from the same vendor in the same way across all laboratories, both in terms of (1) the question the test is asking, which is encoded using the LOINC® standard; and (2) the answer (ie, qualitative result) of the test, which is encoded using the SNOMED CT® standard.^20^ SHIELD’s goal is to achieve cross-institutional laboratory data interoperability by developing a publicly available infrastructure to improve the quality, interoperability, and portability of laboratory data within and between institutions for enabling improvement of patient safety and care, public health reporting, healthcare research and innovation, clinical decision support, regulatory decisions, outbreak monitoring, signal detection, and creation of real-world evidence (RWE).^21^ In 2020, the U.S. Secretary of HHS ordered the use of the LIVD test code mapping for SARS-CoV-2 test results provided by SHIELD.

FDA’s Office of In Vitro Diagnostics and Radiological Health (OIR) within the Center for Devices and Radiological Health (CDRH) funded a SHIELD demonstration program to pilot and evaluate the use of the LIVD standard from IVD manufacturers in five medical center pilot sites.^22^ The objective of this program was to assess the effectiveness of the LIVD specification in medical center laboratory settings and utilize findings gathered to inform future FDA policy on the use of RWE in regulatory decisions.

### Background

Public Law 116–136, § 18115(a) of the CARES Act, requires “every laboratory that performs or analyzes a test that is intended to detect SARS-CoV-2 or to diagnose a possible case of COVID-19” to report the results from each such test to the Secretary of HHS.^23^ In addition, the statute authorizes the Secretary to prescribe the form and manner, and timing and frequency, of such reporting. This document outlines the requirements for data submission to HHS as authorized under this law. The LIVD test code mapping for SARS-CoV-2 test results provides federal direction to laboratories across the nation for IVDs with FDA EUA.^24^

In addition, CMS published an interim final rule (85 FR 54820) requiring all hospitals and critical access hospitals to report information regarding the public health emergency for COVID-19 in accordance with a frequency and in a standardized format as specified by the Secretary. Failure to report the specified data needed to support broader surveillance of COVID-19 resulted in the imposition of financial penalties for a provider’s participation in the Medicare and Medicaid programs.^25^

IVD test results are often represented differently between different institutions, or even within an institution, impacting their utility in patient care, research, and public health use cases. This variation in IVD data results in a lack of interoperability and can increase patient safety risk. To assist, specifications have been developed, such as Laboratory Analytical Workflow (LAW) and LIVD. LAW—developed by the Integrating the Healthcare Enterprise (IHE) Laboratory Technical Committee^26^—is the transport framework for exchanging data between IVD instruments and LIS using Health Level Seven (HL7) V2 messaging standards. LIVD aligns the terminology codes for each IVD by vendor so labs can report such codes properly; it was initially released as a spreadsheet and industry-developed JavaScript Object Notation (JSON) definition and will soon be represented using HL7 Fast Healthcare Interoperability Resources (FHIR®) constructs. FHIR® is the registered trademark of HL7 and is used with the permission of HL7. Other standards are available but not widely implemented to properly exchange data through the continuum of care: inside the lab, between different labs, between providers and labs, between labs and public health institutions, and in access for research.

The objective of the LIVD specification is to define an IVD industry format for use by laboratory personnel or applications to facilitate the publication of LOINC® codes for vendor IVD tests and results. The goal of LIVD is to reduce differences in coding between vendors for similar tests and results and align codes between labs using the same IVD product, leading to semantic consistency of laboratory data. Below is a summary overview of the LIVD specification^22^:

- *Format*—LIVD defines a table and digital format for its data specification. A spreadsheet is recommended as the table format. Spreadsheets can be used to filter the publication content as part of a manual activity to select the LOINC® codes. In addition, table content from multiple vendors can be merged into a single spreadsheet. JSON was selected as the digital format and a LIVD Implementation Guide for integrating the format schema with FHIR® is under development at HL7.^27^
- *Documentation*—The IICC maintains documentation about the data definition and structure of LIVD content.
- *Release* *cycle*—Within the IICC, manufacturers publish their own LIVD catalogs, with varying update frequency (eg, quarterly, annually, as needed).
- *LOINC® and SNOMED CT®*—LOINC® codes and attributes, and optionally SNOMED CT® concepts in some cases, are included within LIVD data catalogs to represent the analytes, specimens, and results for various lab tests.

## OBJECTIVES

The overarching objective of this work was to evaluate the use of the initial SHIELD-approved standards and infrastructure at Implementing Healthcare Institutions (IHI) prior to its consideration on a national scale. The infrastructure intended for implementation consists of:

- Semantic Standards:
  - LOINC®
  - SNOMED CT®
  - UCUM (Unified Code for Units of Measure)^28^
  - UDI (Device Identifier component only)
- Transmission/Mapping:
  - LAW
  - LIVD
  - LIVD FHIR® profile

Because every healthcare institution is different, not all parts of the SHIELD-approved infrastructure are possible or appropriate for all IHIs (eg, institutions not leveraging FHIR® could not implement the LIVD FHIR® profile). The SHIELD evaluation team determined what was reasonable and appropriate for each individual IHI infrastructure to demonstrate interoperability for this study. The specific aims of this work were to:

*Aim 1*: Identify and on-board active healthcare institutions as pilot sites for the assessment of SHIELD-harmonized standards.

*Aim 2*: Collect laboratory test codes to be used in the assessment of implementation of SHIELD-harmonized standards.

*Aim 3*: Evaluate the use of the SHIELD-approved infrastructure at participating IHIs.

## MATERIALS AND METHODS

### Aim 1: Participant recruitment

#### Setting

The pilot program took place from September 2019 to September 2021 in five medical centers’ laboratories across the United States. The program sponsor was FDA’s OIR within CDRH. The pilot program recruitment and evaluation were conducted by Deloitte Consulting LLP. The study was designed to evaluate the implementation of the LIVD file by clinical laboratories during the rollout of the CARES Act reporting requirements for COVID-19. Due to the pandemic, the focus of the study changed to COVID-19 and associated conditions in 2020.

#### Recruitment criteria

To be eligible, medical center laboratories were identified based on their willingness and ability to produce informatics and terminology data from their LIS and health IT systems. The rationale for these priority criteria was to focus efforts on medical center laboratories with some capability to provide RWE given the relatively short 24-month project duration, inform FDA in regulatory decision making, and guide laboratory interoperability rollout on a national scale.

#### Materials and design

A snowball sampling strategy was used to recruit laboratories for this pilot program. We started recruiting from a list of renowned institutions and accepted referrals from initial participants to generate additional candidates. We also sought out potential candidates from SHIELD through presentations at their meetings and email distribution lists. The ideal institution was one that had an individual who was knowledgeable with LIS, was experienced with health IT architecture, and had the time and interest to be a champion for the work.

### Aim 2: Data collection

#### Data collection from medical center laboratory pilot sites

Each medical center was asked to extract about 100 LOINC® codes from their LIS for prioritized tests of interest focused on high-risk conditions and SARS-CoV-2. For each selected test (eg, SARS-CoV-2 RNA COVID-19), we collected the following data elements: test names/descriptions (eg, SARS coronavirus 2 RNA [Presence] in Respiratory specimen by NAA with probe detection), associated instruments (eg, IVD Vendor Model), and LOINC® codes (eg, 94500-6). High-risk conditions were defined by referencing the CDC’s published list of Underlying Medical Conditions Associated with High Risk for Severe COVID-19.^29^ A data collection template spreadsheet was created and disseminated to the medical centers to help provide consistency and reporting clarity for data elements from sites.

#### Data collection from IVD manufacturer

We coordinated with SHIELD stakeholders and the IICC to request manufacturer LIVD catalogs containing the LOINC® codes per IVD instrument per test from manufacturers.

### Aim 3: Evaluation

#### Design

We sought to identify gaps and similarities between LOINC® codes from manufacturer LIVD files versus LOINC® codes from the medical centers. To achieve this aim, the LOINC® codes from manufacturers were compared against LOINC® codes from medical centers for the same tests. We evaluated the gaps and similarities by the two sources (ie, IVD manufacturers vs medical centers) to determine what tests/codes were matched and mismatched.

#### Data cleaning

We cleaned the data submissions from medical center laboratory pilot sites to focus on data points where, at a minimum, instrument name, LOINC® code, and test name were included. We also normalized the instrument names so that they could be compared with the names in the LIVD files provided by manufacturers.

Within a LIVD file, it may be acceptable for manufacturers to not specify LOINC® codes for every test because such LOINC® codes may not exist to address the data reporting needs of the given test. However, for analysis and comparison purposes, we focused on the data points where LOINC® codes were provided.

#### LOINC® comparison: Matches

We identified medical center and manufacturer intersections where a medical center used an instrument that was present in the LIVD file sent to us by a particular manufacturer. Then, we ran a query in a SQL database for every combination of medical center and manufacturer where the Instrument Name in the medical center data set corresponded to an Instrument Name in the LIVD file and where the LOINC® code matched.

#### LOINC® comparison: Mismatches

We identified every record in the data submitted by the medical centers that did not have a match for the corresponding manufacturer LIVD file. This was done for each intersection in the medical center versus manufacturer data. For each record where there was a recorded mismatch, we manually verified whether or not there were any close matches by having a health informatics/terminology specialist verify the integrity of each mismatch and close match.

## RESULTS

### Aim 1: Participant recruitment

We identified 28 medical center laboratories in the United States to gauge interest and recruit participants for this SHIELD pilot program: six declined to participate due to the COVID-19 pandemic and lack of interest in terminology standards, while 17 expressed initial interest. Ultimately, we moved forward with recruiting five pilot sites for the full pilot program. Reasons for laboratories dropping from consideration to participate in the study included: (1) response not received after initial communications, (2) limited bandwidth, and (3) site leadership did not feel that they could support the program’s scope of work. In addition, the period of performance for this body of work coincided with the COVID-19 pandemic and laboratory sites had an increasing number of competing priorities.

We worked with an analytic sample of five medical centers. The medical centers ranged in size from 553 to 1841 hospital beds from five distinct states (Connecticut, Florida, Maryland, Nebraska, and Utah). The participating medical centers were: Intermountain Healthcare, Johns Hopkins University, Yale University, University of Nebraska Medical Center, and University of Miami.

### Aim 2: Data collection

**Figure 1.**
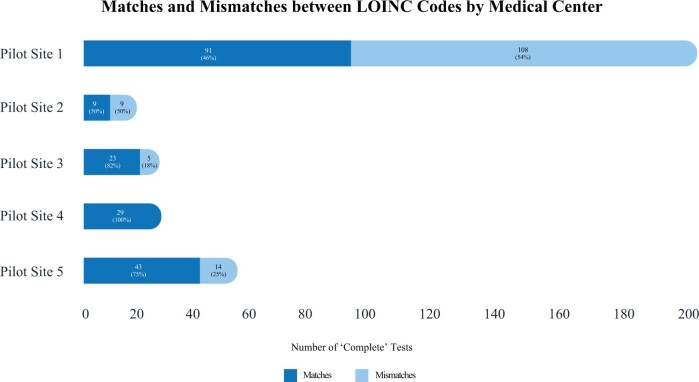
Medical center pilot site interoperability matches and mismatches between manufacturers and medical centers.

Five medical centers extracted data from their LIS for prioritized tests of interest focused on high-risk conditions and SARS-CoV-2. The data submission ranged from 74 LOINC® codes to 532 LOINC® codes per site. Three IVD Manufacturers (Abbot, BioMérieux, and Roche) submitted 15 LIVD catalogs representing 26 distinct devices, 6956 tests, and 686 LOINC® codes (this included test data from SHIELD’s COVID-19 LIVD file for these three manufacturers). Table 1 summarizes the data collected from the pilot sites and the matches/mismatches between LOINC® codes from the manufacturer LIVD files versus LOINC® codes from the medical centers. Figure 1 shows the breakdown of matches and mismatches.

**Table 1.** Medical center pilot site data reporting including interoperability matches and mismatches between manufacturers and medical centers

Medical center pilot site	Tests collected	Tests considered “complete”^a^	“Complete”^a^ tests available in LIVD files	LOINC® matches	LOINC® mismatches
Non-COVID-19 tests					
Pilot Site 1	520	508	197	91	106
Pilot Site 2	66	59	13	5	8
Pilot Site 3	72	66	22	17	5
Pilot Site 4	532	526	29	29	N/A
Pilot Site 5	131	112	56	42	14
Non-COVID-19 total	1321	1271	317	184	133
COVID-19 tests					
Pilot Site 1	2	2	2	0	2
Pilot Site 2	8	8	5	4	1
Pilot Site 3	8	8	6	6	0
Pilot Site 4	0	0	0	0	0
Pilot Site 5	8	1	1	1	0
COVID-19 total	26	19	14	11	3

a “Complete” tests contained an instrument name, LOINC® code, and test name.

### Aim 3: Evaluation

We identified mismatches in how medical centers used LOINC® to encode laboratory tests compared to how IVD manufacturers used LOINC® to encode the same laboratory tests in the LIVD catalogs. Of 331 tests available in the LIVD files, 136 (41%) were represented by a mismatched LOINC® code by the medical centers. Even with this small sample size, the variation in the performance of these medical centers is notable; a chi-square test of the hypothesis that the rates of agreement are equal is rejected (chi-square 45.0, 4 df, *P* < .0001).

#### LOINC® comparison: Matches

We identified 195 LOINC® codes (59%) in the manufacturer LIVD files that were matches with LOINC® codes used at pilot site medical centers for the same tests. Table 2 shows a representative sample of these matches.

**Table 2.** Manufacturer recommended LOINC® codes that match LOINC® codes used in medical center pilot sites^a^

Site	IVD Manufacturer and device name	LOINC® code	LOINC® long common name	LIS test name
Pilot Site 1	Roche Cobas	1751-7	Albumin [Mass/volume] in Serum or Plasma	Albumin
		17862-4	Calcium [Mass/volume] in Urine	Calcium, urine random
		2350-7	Glucose [Mass/volume] in Urine	Glucose, urine
		2339-0	Glucose [Mass/volume] in Blood	Glucose, pregnancy screen
Pilot Site 2	Abbott Architect i2000	33935-8	Cyclic citrullinated peptide IgG Ab [Units/volume] in Serum	Cyclic citrul peptide Ab, IgG
		13950-1	Hepatitis A virus IgM Ab [Presence] in Serum or Plasma by Immunoassay	Hepatitis A Ab, IgM
		24113-3	Hepatitis B virus core IgM Ab [Presence] in Serum or Plasma by Immunoassay	Hepatitis B core Ab, IgM
	BioMérieux Biofire Torch	94565-9	SARS coronavirus 2 RNA [Presence] in Nasopharynx by NAA with non-probe detection	SARS-CoV-2 by PCR (Biofire)
Pilot Site 3	Roche Cobas c 702	2160-0	Creatinine [Mass/volume] in Serum or Plasma	Creatinine, blood
		2324-2	Gamma glutamyl transferase [Enzymatic activity/volume] in Serum or Plasma	GGTP
		1975-2	Bilirubin.total [Mass/volume] in Serum or Plasma	Total bilirubin
		2885-2	Protein [Mass/volume] in Serum or Plasma	Total protein
Pilot Site 4	BioMérieux FilmArray	82207-2	Entamoeba histolytica DNA [Presence] in Stool by NAA with non-probe detection	Entamoeba histolytica DNA
		82199-1	Salmonella enterica+bongori DNA [Presence] in Stool by NAA with non-probe detection	Salmonella enterica+bongori DNA
		82190-0	Herpes simplex virus 1 DNA [Presence] in Cerebral spinal fluid by NAA with non-probe detection	Herpes simplex virus 1 DNA
		82190-8	Herpes simplex virus 2 DNA [Presence] in Cerebral spinal fluid by NAA with non-probe detection	Herpes simplex virus 2 DNA
Pilot Site 5	Roche Cobas 8000/ISE Module	2823-3	Potassium [Moles/volume] in Serum or Plasma	Potassium
	Roche Cobas e 601	6598-7	Troponin T.cardiac [Mass/volume] in Serum or Plasma	Troponin T
	Roche Cobas c 702	2571-8	Triglyceride [Mass/volume] in Serum or Plasma	Triglycerides
	Roche Cobas c 502	2458-8	IgA [Mass/volume] in Serum or Plasma	Immunoglobulin A

a We identified 195 LOINC® codes in the manufacturer LIVD files that were matches with LOINC® codes used at pilot site medical centers for the same tests. This table shows a representative sample of these matches.

#### LOINC® comparison: Mismatches

We identified 136 LOINC® codes (41%) in the manufacturer LIVD files that were mismatches with LOINC® codes used at pilot site medical centers. Table 3 shows a representative sample of these mismatches.

**Table 3.** Manufacturer recommended LOINC® codes that do not match LOINC® codes used in medical center pilot sites^a^

Site	IVD manufacturer and device name	Pilot site data			Manufacturer data for similar test		Potential reason for mismatch
		LIS test name	LOINC®	Pilot test description	LOINC®	LOINC® long common name	
Pilot Site 1	Roche Cobas	Calcium, urine	18488-7	Calcium	17862-4	Calcium [Mass/volume] in Urine	LOINC® is for 24 h urine –the IVD manufacturer/instrument may not be aware
		Cannabinoid screen	70143-3	Cannabinoids	8172-9	Cannabinoids [Presence] in Serum or Plasma by Screen method	Quantitative LOINC® vs qualitative by the manufacturer
		Prolactin, serum	2842-3	Prolactin	20568-2	Prolactin [Mass/volume] in Serum or Plasma by Immunoassay	Methodless LOINC®—still a valid LOINC®, but not as granular as the manufacturer LOINC®
Pilot Site 2	Abbott Architect i2000	Hepatitis C Ab	16128-1	Hepatitis C virus Ab [Presence] in Serum	13955-0	Hepatitis C virus Ab [Presence] in Serum or Plasma by Immunoassay	Methodless LOINC®—still a valid LOINC®, but not as granular as the manufacturer LOINC®
		HBsAb, quantitative	16935-9	Hepatitis B virus surface Ab:ACnc:Pt:Ser:Qn	5193-8	Hepatitis B virus surface Ab [Presence] in Serum by Immunoassay	Quantitative LOINC® vs qualitative by the manufacturer
Pilot Site 3	Roche Cobas c 702	Blood urea nitrogen	3094-0	BUN	14937-7	Urea nitrogen [Moles/volume] in Serum or Plasma	Different units of measure: mass/volume versus moles/volume
		AST (SGOT)	1920-8	Aspartate aminotransferase (AST)	30239-8	Aspartate aminotransferase [Enzymatic activity/volume] in Serum or Plasma by With P-5'-P	Methodless LOINC®—still a valid LOINC®, but not as granular as the manufacturer LOINC®
Pilot Site 5	Roche Cobas c 702	BUN	3094-0	Urea nitrogen [Mass/volume] in Serum or Plasma	14937-7	Urea nitrogen [Moles/volume] in Serum or Plasma	Different units of measure: mass/volume versus moles/volume
		Aspartate aminotransferase (AST)	1920-8	Aspartate aminotransferase [Enzymatic activity/volume] in Serum or Plasma	30239-8	Aspartate aminotransferase [Enzymatic activity/volume] in Serum or Plasma by With P-5'-P	Methodless LOINC®—still a valid LOINC®, but not as granular as the manufacturer LOINC®

a We identified 136 LOINC® codes in the manufacturer LIVD files that mismatch LOINC® codes used at pilot site medical centers. This table shows a representative sample of these mismatches.

## DISCUSSION

The five medical centers varied in how they organized, categorized, and stored LIS catalog information. This variation impacts data quality and interoperability. A summary of key findings includes the following:

1. Medical center LIS test catalogs included data quality inaccuracies with LOINC®, such as using codes that were not proper LOINC® codes, deprecated LOINC® codes, discouraged LOINC® codes, and trial LOINC® codes.
2. Medical center LIS catalogs contained duplicative information (repeated tests) or tests that changed meaning over time (internal ID or description changed over time).
3. Medical centers expressed that the LIVD catalog is helpful as a centralized platform, taking away LOINC® guesswork and reducing LOINC® variation between systems.
4. The LIVD catalog is helpful in the selection of LOINC® codes associated with specific COVID-19 testing platforms.
5. There were minor inconsistencies with how different manufacturers organized and stored IVD test information within LIVD catalogs likely due to not using the same version of the LIVD standard (eg, slightly different column names for the same data elements).
6. There is potential for the LIVD catalogs to help improve semantic interoperability and data quality of LIS data; there is also room for improvement of the LIVD catalog data elements and accessibility of LIVD files by labs.

## CONCLUSION

### Limitations of this study

#### Small participant size

In the selection criteria for pilot sites, we did not attempt to recruit a sample of laboratories that were representative of the entire United States. Superior IT and IT capabilities around data interoperability were the selection criteria.

#### Data collection limitations

Even with this small sample of 331 tests from five sites, variation exists in how the data were encoded. Within and across the pilot sites, there is a lack of standardization for encoding test data. To promote interoperability at the national level for laboratories across the country, there is a need for continuing and additional efforts for standardization.

Data submitted by Pilot Site 1 did not include the specific model for IVD instruments from the Roche manufacturer. As a result, all of the Pilot Site 1 versus Roche matches and mismatches are general matches and not specific matches. For all other matches and mismatches, data provided by the pilot sites and data provided by the manufacturers can be compared directly since we had the specific models for the IVDs.

This study only includes LIVD catalogs from three IVD manufacturers. Three of the pilot sites had a low number of tests (< 30) performed on instruments from the three participant manufacturers in the study. Furthermore, we did not require the use of LIVD prior to assignment of LOINC® codes to their tests.

This program did not consider laboratory developed tests (LDTs). Insights from additional laboratories for an increased number of tests are needed to make the findings more comprehensive.

#### Inconsistencies in underlying standards

The scope of work did not address inconsistencies or gaps in the LOINC® and SNOMED CT® terminologies that are used within the LIVD specification files. As new tests are created and rolled out to laboratories, there is an opportunity for LIVD to standardize and harmonize how these tests are represented, thereby remediating overlaps and inconsistencies that may exist as LOINC® and SNOMED CT® codes are applied across medical centers and used by IVD vendors in their LIVD files.

#### Current LIVD limitations

There are limitations to the use of LIVD in its current state of development to improve laboratory data interoperability.

The COVID-19 LIVD file is curated by a SHIELD committee and hosted by CDC for distribution. A potential limitation is whether SHIELD is representative of all stakeholders and interests and whether there should be an independent validation of the COVID-19 LIVD file content.

The LIVD file format is owned by IICC, and the JSON representation is a project at HL7. The LIVD file is a spreadsheet distributed by IVD manufacturers to their customers; the distribution, installation, and searching of LIVD catalogs is not yet automated in the SHIELD and laboratory system.

A potential limitation is that the LIVD standard was perhaps not well known or used prior to COVID-19. The mechanism of LIVD started to take hold for COVID-19 due to CMS reporting requirements and the dynamism of the pandemic with EUA devices, forcing laboratories to seek out a “source of truth.” CMS/CDC directed labs to LIVD, and in this study we found better level of agreement in LOINC® coding specific to COVID-19 (9 out of 11 matches).

### Suggestions for future work

To facilitate widespread adoption of LIVD, there is a need to continue demonstrating LIVD’s value and establish LIVD in the workflows of medical centers, LIS/LIMS interfaces, and HL7 messages. There is an opportunity to further pursue and build out the use cases for LIVD by engaging an increasing number of medical center laboratories and obtaining their feedback so that LIVD can be used in laboratory health IT environments. A formative evaluation of LIVD use cases should be an ongoing effort to demonstrate LIVD’s value. This should be part of a comprehensive system of quality control built into the system.

Furthermore, there is a need to update the format of LIVD to make it more accessible by providing tooling and including proper value sets for specimen type, results when qualitative, coded units of measures for quantitative results, and additional metadata. It would also be beneficial to have a repository of LIVD files from different manufacturers.

Suggested next steps include additional collaboration among key players in the laboratory ecosystem, including standards development organizations, device manufacturers, and medical centers. Enhanced program support for SHIELD is necessary to effectively roll out LIVD in medical centers and promote laboratory data interoperability nationally.

This work provides tools and approaches that may be useful in the development of a national evaluation of implementation of harmonized SHIELD standards. Case studies are proposed as part of an evaluation framework in the SHIELD Strategic Plan.

### Implication of future implementation of harmonized coding for IVD data

This report documents the potential effects of the HHS order regarding laboratory data reporting intended to accomplish interoperability in five leading medical center laboratories. Even within institutions with sophisticated IT and LIS environments, there may be an incomplete understanding of LOINC®. Communication of the availability, centralized distribution, and support for LIVD files and harmonized standards is needed to support laboratory informatics infrastructure and capacity to respond to analyze data. The pilot study indicates that a more comprehensive policy and program is needed to achieve laboratory interoperability.

## FUNDING

This work was supported by the U.S. Food and Drug Administration Office of In Vitro Diagnostics and Radiological Health within the Center for Devices and Radiological Health Contract # 75F40119C10164, “Implementation of SHIELD Harmonized Laboratory Data Standards in Healthcare Institutions.”

## AUTHOR CONTRIBUTIONS

MSW, GP, WSR, and SAB conceived the study idea. RAC, GP, EDK, GR, AKS, IKA, and NMS wrote the initial manuscript and collected the data from the pilot sites. IKA and EDK performed the data analysis. RM performed a detailed review of the LOINC matches and mismatches. WCH performed the statistical analysis. All authors revised and expanded the manuscript. All authors reviewed and approved the manuscript prior to submission.

## DISCLAIMER

This article reflects the views of the authors and should not be construed to represent FDA’s views or policies.
